# Poly-γ-glutamic acid enhances the quality of recombant erythropoietin produced by CHO cells

**DOI:** 10.1080/13102818.2014.901675

**Published:** 2014-07-18

**Authors:** Tae Gon Kim, Young Chol Cho, Bok-Hwan Chun, Sung Hyo Park, Hoi-Seon Lee, Namhyun Chung

**Affiliations:** ^a^ College of Life Sciences and Biotechnology, Korea University, Seoul, Korea; ^b^ College of Agriculture and Life Science, Chonbuk National University, Jeonju, Korea

**Keywords:** additives, chemically defined medium, CHO cells, EPO-dependent F-36E cells, Pluronic F68, poly-γ-glutamic acid, recombinant erythropoietin

## Abstract

The effect of poly-γ-glutamic acid (γPGA), which is produced by Bacillus sp., on the production of recombinant erythropoietin (rEPO) by Chinese hamster ovary (CHO) cells in suspension culture was evaluated. The growth, viability, and productivity of recombinant CHO cells were investigated in a chemically defined medium with 50 and 500 kD γPGAs at 0.075% or with Pluronic F68. Cell growth with the two γPGAs was lower than that with Pluronic F68 but significantly higher than that without any additive (control). The effect of additives on rEPO productivity was 50 kDa γPGA > 500 kDa γPGA > Pluronic F68 > control. Using EPO-dependent F-36E cells, we found that the effect of additives on rEPO quality was 500 kDa γPGA > 50 kDa γPGA > control > Pluronic F68. γPGA has an enhancement effect on the quality of rEPO produced by CHO cells.

## Introduction

Cultivation of mammalian cells producing recombinant therapeutic protein is important for the biopharmaceutical market.[1] Chinese hamster ovary (CHO) cells are commonly used mammalian cells for large-scale production of recombinant proteins. Since CHO cells offer stable and accurate glycosylation, they provide more accurate processing of natural proteins *in vitro*.[2] From an industrial perspective, the ability of CHO cells to adapt and grow in suspension is highly desirable as it allows volumetric scalability and use of large stirred-tank bioreactors with a serum-free medium.[3] Mammalian cells are particularly fragile; however, the presence of certain chemical additives (i.e., serum, pluronic alcohols, bovine serum albumin, and polyethylene glycol) can effectively reduce animal cell damage in suspension culture.[4] The most widely used protective medium additive, Pluronic F68, has been proven to support high-densities suspension culture of animal cells over time.[5] However, many problems have been reported with Pluronic F68, including reduced growth rate due to the lower rate of DNA synthesis in continuous and short-term cultures,[6] as well as decreased recombinant protein productivity under sublytic levels of shear stress.[7] For these reasons, other efficient medium additives to replace and/or complement Pluronic F68 need to be investigated.[8]

Erythropoietin (EPO) is a 30.4 kDa glycoprotein and a haemopoietic hormone, which primarily stimulates the proliferation and differentiation of erythroid precursor cells in the treatment of renal and nonrenal anaemias.[9] Poly-γ-glutamic acid (γPGA) is the most promising biodegradable polymer that is produced by *Bacillus subtilis*. γPGA is well characterized as water soluble, biodegradable, edible, and nontoxic in humans and the environment.[10] Previously, we have shown that γPGA could be an attractive alternative polymer for use as a medium additive to avoid shear stress damage during the cultivation of CHO DG44 cells.[11] However, it was not clear how the presence of γPGA affects the production and quality of proteins by CHO cells. In this study, we compared the effect of γPGA and Pluronic F68 medium additives on the productivity and quality enhancement of recombinant erythropoietin (rEPO) produced by CHO cells in a chemically defined medium.

## Materials and methods

### Cell line and cell culture

The recombinant human EPO-producing CHO cell line (EC2-2C5; rCHO) constructed by transfecting the cDNA encoding human EPO under the control of the cytomegalovirus promoter into DG44 CHO cells was kindly provided by Jung-Hoe Kim, KAIST (Taejon, Korea). The EC2-2C5 cell line was cultured as a monolayer in 14 mL of MEMα modification medium (WelGENE, Daegu, Korea) supplemented with 10% (v/v) dialysed FBS (SAFC Bioscience, Lenexa, KS, USA) and 20 nmol/L MTX in 75 cm^2^ T-flasks. The cells were incubated at 37 °C in a humidified 5% CO_2_ atmosphere. Cell viability was determined by trypan blue dye exclusion with a haemocytometer.

### Adaptation in suspension

The cells were adapted directly to suspension growth in a chemically defined medium for CHO cells (HyQCDM4CHO, Hyclone, Logan, UT, USA) supplemented with 2 nmol/L MTX in 125 mL Erlenmeyer flasks on a rotary shaker at 110 r/min. To investigate the effect of polymer additives, 30 mL of culture medium was supplemented with Pluronic F68 or γPGA at each concentration. For maintenance, cells were seeded at a concentration of 3 × 10^5^ cells/mL in fresh medium every 3 days. The growth rate (h^−1^) was calculated as follows[12]: μ = (1/*x*)(d*x*/d*t*), where μ is the growth rate, d*x* is the increase in cell number, d*t* is the time interval, and *x* is the number of cells.

### Preparation of medium additives

The medium additives were Pluronic F68, 50 kDa γPGA, and 500 kDa γPGA. Pluronic F68 was purchased from Sigma-Aldrich Chemical Co. (St. Louis, MO, USA) as a 10% (w/v) stock solution, and 50 kDa and 500 kDa γPGAs were supplied by Bioleaders (Daejeon, Korea). Each γPGA polymer powder was dissolved in HyQCDM4CHO medium at a concentration of 1% (w/v), and filtered with a 0.22 μm membrane, and adjusted to a pH of 7.0–7.2. The stock solutions were diluted in HyQCDM4CHO medium at the final concentrations of 0.05%, 0.075%, and 0.1%.

### Measurement of rEPO production by immune assay

To measure the concentration of rEPO, 1 mL of culture media was collected from each culture suspension and centrifuged at 2500× g for 5 min to obtain the supernatants. The expression levels of secreted rEPO were measured by Quantikine Human EPO Immunoassay kit (R&D Systems, Minneapolis, MN, USA) according to the supplier's instructions.

### Bioassay of rEPO

The biological activity of rEPO in the culture medium was evaluated using F-36E, an EPO-dependent cell line, derived from the human bone marrow (RCB0776, RIKEN Cell Bank, Ibaraki, Japan). F-36E cells were maintained in RPMI 1640 (WelGENE) in the presence of EPO.[13] The supernatant from the culture medium with γPGAs or Pluronic F68 was frozen at −20 °C before analysis. F-36E cells were inoculated at a cell density of 1 × 10^4^ cells per well in 96-well plates without EPO; the supernatant was then added to each well. After incubation for 3 days, cell viability was measured by the WST-1 assay.

## Results and discussion

### Adaptation of rCHO cells in suspension culture with polymer additives

**Figure 1.**
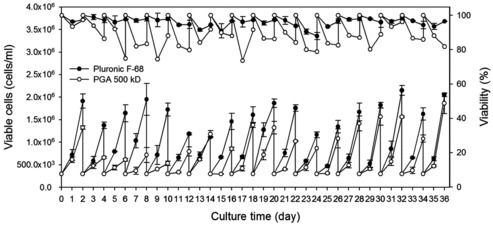
Growth and viability profile of rCHO cells during adaptation in suspension with Pluronic F68 and γPGA. Cells were adapted to 0.1% (w/v) of Pluronic F68 (•) and 0.075% (w/v) of γPGA 500 kD (○). Values indicate mean ± SD of triplicate cultures. The upper part of the graph is for per cent of viability and the lower part is for viable cells density.

The EPO-producing CHO cell line (rCHO) had been already adapted to Pluronic F68 during its development. When a new medium additive is introduced, CHO cells need to adapt to the new medium additive. Therefore, rCHO was adapted to 500 kDa γPGA at 0.075% for 36 days (Figure 1). The viable cell number with γPGA was about 33%–70% lower than that with 0.1% Pluronic F68 before day 16. However, the cell number with γPGA gradually increased to >80% of the cell number with Pluronic F68 after day 16. The viability of rCHO cells with PGA was approximately 90% on average of the viability with Pluronic F68.

**Figure 2.**
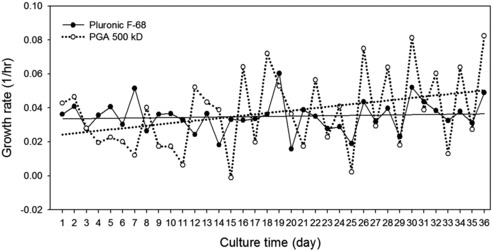
Growth kinetics of rCHO cells during adaptation in suspension with Pluronic F68 and γPGA. Solid line indicates the trend of growth rate with Pluronic F68 and dotted line the trend of growth rate with γPGA.

The effect of each polymer additive on the growth kinetics is shown in Figure 2. Before day 16 of rCHO cell culture with γPGA, the growth rate of 0.027 h^−1^ (doubling time [*T_d_*] = 25.6 h) was lower than that of 0.035 h^−1^ with Pluronic F68 (*T_d_* = 19.9 h) on average. After day 16, the growth rate of rCHO cells with γPGA was improved to 0.045 h^−1^ on average (*T_d_* = 15.5 h). The trend line of the cell growth with γPGA (solid line, Figure 2) increased continually, but the cell growth with Pluronic F68 (dotted line, Figure 2) was almost constant (Figure 2).

### Effect of γPGA concentrations on cell growth and viability of rCHO cell suspension culture

**Figure 3.**
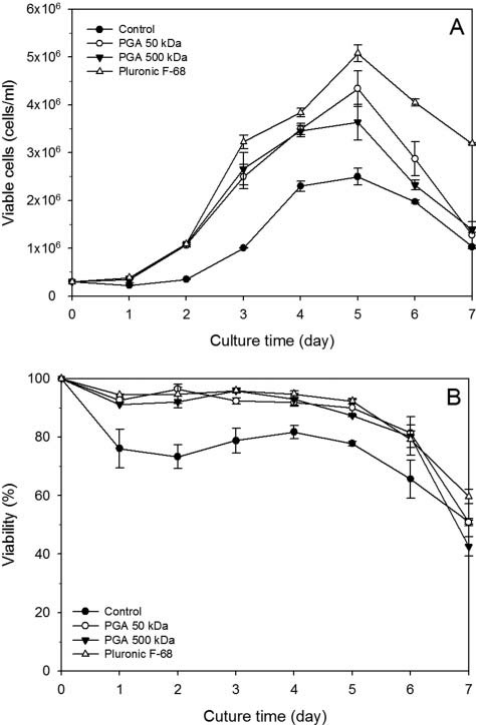
Growth (A) and viability (B) profiles of rCHO cells in HyQCDM4CHO with Pluronic F68 and γPGAs: 0.075% of 50 kDa γPGA (○), 0.075% of 500 kDa γPGA (▴), and 0.1% of Pluronic F68 (△) were added to the culture medium. Control (•) had no medium additive in the culture medium. Values indicate mean ± SD of triplicate cultures.

To evaluate the effect of various concentrations of 50 and 500 kDa γPGAs on the protection of rCHO cells from shear stress, cell growth was investigated using a concentration of 0.05%, 0.075%, and 0.1% γPGAs. Both 50 and 500 kDa γPGAs at different concentrations favoured cell growth in the order of 0.075% > 0.05% > 0.1% (data not shown). No difference in cell growth was observed between 50 and 500 kD γPGA except on days 5 and 6 at 0.075% (Figure 3(A)).

### Effect of medium additives on cell growth and viability

To compare the effect of γPGAs and Pluronic F68, the cell growth and viability were measured in the presence of no additive (control), 50 and 500 kDa γPGAs at 0.075%, and 0.1% Pluronic F68 (Figure 3(A)). On day 5, the cell density with Pluronic F68, 50 kDa PGAs, 500 kDa γPGAs, and control was 5.1 × 10^6^, 4.3 × 10^6^, 3.7 × 10^6^, and 2.5 × 10^6^ cells/mL, respectively. Therefore, Pluronic F68 supported a higher cell density than γPGAs; however, γPGAs also supported a higher cell density than the control medium. This result is in contrast with our previous results with γPGAs, which demonstrated that γPGAs supported cell growth similar to Pluronic F68.[11] The difference is possibly due to the different CHO cell lineage. The viability of rCHO cells was similar among Pluronic F68 and both γPGAs, but lower in the control during cell growth (Figure 3(B)).

### Effect of medium additives on rEPO production

**Figure 4.**
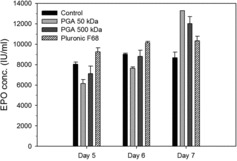
Production level of recombinant erythropoietin in the culture medium with medium additives. Samples of rCHO cell culture were obtained from each medium on days 5–7. The rEPO concentration was expressed as IU (international unit), according to the WHO standard 87/684 (recombinant human EPO). Values indicate mean ± SD of triplicate cultures.

The effect of 0.075% γPGAs and 0.1% Pluronic F68 on the productivity of rCHO cells was investigated. To assay the cumulative concentration of rEPO, samples of the cell culture were obtained from each culture supernatant on days 5–7 (Figure 4). On day 5, rEPO concentrations with control, 50 kDa γPGAs, 500 kDa γPGAs, and Pluronic F68 were 8055, 6152, 7110, and 9.251 IU/mL, respectively. On day 6, rEPO concentrations with γPGAs of 50 and 500 kDa were 7646 and 8813 IU/mL, respectively. These values were lower than those of the control and Pluronic F68. However, on day 7, rEPO concentrations with 50 and 500 kDa γPGAs were 13,275 and 12,020 IU/mL – the values were 125% and 116% of Pluronic F68, respectively. Essentially no difference in the rEPO concentrations was observed between days 6 and 7 in the control or with Pluronic F68. The concentration of rEPO produced by the medium additives and the control on day 7 was in the order of 50 kDa γPGA > 500 kDa γPGA > Pluronic F68 > control (Figure 4).

### Biological activity of rEPO produced in the presence of medium additives

**Figure 5.**
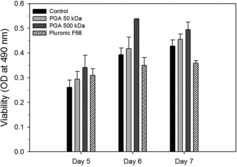
Viability of F-36E cells in the presence of culture media containing rEPO. The culture media containing rEPO was added to the culture of F-36E cells, EPO-dependent human leukaemic cells. WST-1 assay was measured at 490 nm. Values indicate mean ± SD of triplicate cultures.

The biological activity of rEPO with different culture media from days 6 and 7 was investigated using F-36E cells (Figure 5). The F-36E cell viabilities (i.e., biological activities) were measured with culture media containing Pluronic F68 and 50 or 500 kDa γPGAs. The viabilities of F-36E cells with culture media containing γPGAs were higher than those with control and Pluronic F68. The viabilities with culture media containing 50 and 500 kDa γPGAs on day 6 were 114% and 127% of those in the control, respectively. The effect of medium additives on rEPO biological activities was in the order of 500 kDa γPGA > 50 kDa γPGA > control > Pluronic F68. Overall, the rEPO biological activities increased on day 7 but the difference in the biological activities decreased. Unexpectedly, the viabilities with Pluronic F68 were 89% and 84% of the control on days 6 and 7, respectively. These results showed that although growth with γPGAs was less than that with Pluronic F68, the concentration (with the exception of day 6) and biological activity of rEPO with γPGAs were significantly higher than those with Pluronic F68.

### Final remarks

In this study, two γPGAs of 50 and 500 kDa were employed to find the optimal molecular weight and concentration of γPGAs. The results showed that the cell growth was dependent upon both molecular weight and concentration of γPGAs for the productivity and biological activity of rEPO. Regardless of our positive results with γPGA in the production of rEPO, further studies are required for *in situ* application of γPGA, such as its application in large stirred-tank bioreactors using serum-free medium and in downstream processing such as harvest and purification. Our findings with γPGA may be applicable to other biopharmaceuticals.

## Conclusions

To the best of our knowledge, we showed for the first time that a biologics, rEPO, can be produced by CHO cells in the presence of γPGA in place of Pluronic F68, and that CHO cells can be adapted to the presence of γPGA to be protected from shear stress during production of pharmaceuticals. Although Pluronic F68 gave a little higher CHO cells growth and amount of produced rEPO than γPGA, the quality of rEPO as measured by viability assay was higher with γPGA than with Pluronic F68. This also suggests that the use of γPGA instead of Pluronic F68 may be a suitable alternative to enhance the quality of rEPO produced by CHO cell suspension cultures.
